# Putting “Heavy” into Heavy Slow Resistance

**DOI:** 10.1007/s40279-022-01641-y

**Published:** 2022-01-27

**Authors:** Scot Morrison, Jill Cook

**Affiliations:** 1 Physio Praxis PLLC, Vancouver, WA USA; 2 Musculoskeletal Research Centre at La Trobe University, Melbourne, Australia; 3 Department of Neuroscience, Biomedicine and Movement Sciences, University of Verona, Verona, Italy

## Abstract

The body of literature on tendinopathy management has come a long way in the last few decades and a variety of changes in the clinical approach have emerged from this research. One particular approach that shows promise has been called “heavy slow resistance” (HSR), and this has been the subject of investigation in a number of randomized controlled trials. While the premise for this approach is defensible, a critical examination of the implementation of these HSR protocols results in some concerns when compared to basic exercise science principles. This article lays out some considerations that will help future investigators to improve their exercise prescription approaches in this area.

## Key Points

**Table:** 

Relative submaximal lifting capacity (RSLC) will dictate repetitions at a set load and slower tempos compound this effect.	
Heavy slow resistance (HSR) is commonly used when managing tendinopathies since tendon strain sufficient for adaptation is generally more likely at a higher percentage (> 70%) of maximum, but the RSLC will limit total repetitions possible at that load (< 6).	
This means that volume must derive from something other than increasing repetitions per set if %RM and tempo are maintained.	

## Introduction

The management of tendon pain via progressive loading is well documented and appears to be one of the best interventions currently available. The traditional emphasis placed on eccentric contractions does appear to be unnecessary though, since similar outcomes are seen regardless of contraction type, and so heavy slow resistance (HSR) has become an accepted approach for the early stages of management due, in part, to its ability to moderate the rate of loading while still providing a sufficient load [1–5]. While uncertainty surrounds the reason for these improvements (e.g., adaptations in some or all of tendon, muscle, kinetic chain and/or brain), patient outcomes do support the use of this intervention. However, there are some fundamental programming principles that appear to be missed in the HSR literature that require clarification in order to make exercise prescription match the idea contained in the title “HSR.”

## Background

Targeted exercise prescription has a variety of parameters that influence each other and can be manipulated to influence the desired outcomes. These include the rate of loading (slope of the force/time curve), the intensity (load as % of maximum), the time under tension, the rest between sets, the individual’s perceived effort, the joint positions and muscle lengths during loading, and the volume-load (load x sets x repetitions) performed. Of these, the exercise intensity and rate of loading are two of the most important parameters prescribed to address tendon issues. While this paper focuses on HSR training, sports with significant elastic energy storage demands require rehabilitation that address these demands, and while it is traditionally addressed after a HSR program, some concurrent work could be indicated.

To create change in the mechanical properties of the tendon, stress must be applied to the tendon at a magnitude that causes sufficient strain [6]. Practically speaking, a loading approach at higher intensities, defined by percentage of one repetition maximum [1RM, the maximum weight that can be lifted once (%RM)], is generally an acceptable proxy for this. For example, a systematic review by Bohm et al. found the intensity threshold likely to ensure adaptations in mechanical, material, and possibly morphological properties of tendon was 70% of maximum, and this seems to be true regardless of contraction type [7, 8]. It is important to remember that this 70% RM is a general estimate of a continuous variable whose true value will vary based on the individual’s muscular strength relative to the stiffness of their tendons. This is evidenced by findings like those of Quinlan et al., reporting tendon changes at a lower load (60% RM), and others reporting upwards of 90% of RM being necessary to see sufficient strain [6, 9]. Because of this, in a clinical setting it may be better to view prescriptions based on loads of > 70% as a “good starting bet” to increase the probability of achieving the necessary tendon strain for adaptation.

In a high-performance setting a 1RM can be determined by lifting progressively heavier loads until a one-rep-max is reached, but clinically this may be contraindicated. Alternatively, a 1RM can be estimated by assessing a submaximal load taken to the point where another repetition is not possible (i.e., performing a 3RM or a 5RM to calculate a 1RM). The accuracy of the calculated 1RM is inversely related to the number of reps achieved. Therefore, the load lifted should be low enough to hit at least two to three repetitions, but failure occurring at or before eight repetitions is likely best. All testing should be done after a warm-up of progressively heavier loads with sufficient rest between efforts to prepare the individual and potentially minimize the impact that pain and fatigue have on the test.

## Heavy Slow Resistance Considerations

### Relative Submaximal Lifting Capacity

Individual factors influence the ability to attain sufficient intensity in HSR training. The training background of the person is critical to understanding their RSLC. For example, total repetitions achieved on the leg press at 70% RM varied widely based on training history: individuals who trained for endurance completed 39.9 (± 17.6 reps) compared to those who trained for strength (17.9 ± 2.8 reps) [10]. The ability to complete repetitions at a given %RM has been termed “relative submaximal lifting capacity” (RSLC). This RSLC is influenced by a variety of other factors as well, such as the training status of the individual with trained individuals managing 9.67 (± 0.91) repetitions compared with untrained people (7.14 ± 0.74) when tested at 85% of 1RM in the back squat [11]. This is important to consider in tendon conditions, as some tendinopathies occur in highly trained people (e.g., patellar tendinopathy), while others can occur in more sedentary populations (e.g., gluteal tendinopathy).

In addition, the relationship of the RSLC to the 1RM varies across time in individuals. Braith et al. investigated 1RM and 7–10RM in a training group over time, and found that the % of RM increased more for the 7–10RM test than the max did [12]. The 7–10 repetition range increased by more than 10% (pre-training 68.4% ± 7.2 of RM baseline, post-training 79.1% ± 7.0 of RM). While the predictive ability of this 7–10RM test for 1RM was good (± 10%), it overestimated the RM in trained individuals, which suggests a more accurate estimating procedure that is based on training state may need to be developed. It can be concluded then that fluctuations in RSLC based on training type and status will influence the actual %RM intensity used even if repetition ranges are similar. Despite this variability, if 12 repetitions or more can be completed with a given load it is highly likely that this load will be less than the 70% RM intensity goal of tendon loading.

Of course, these studies have all involved uninjured individuals, and in a patient population the length of tendon symptoms and the resulting dysfunction may influence the relative intensity as well. Tendon pain typically results in a person unloading the affected area during tasks, which means most multi-joint and bilateral exercises find the individual using a movement strategy that decreases the load on the affected tendon while still accomplishing the task. This ability to offload the tendon can be controlled through the use of isolated exercises, as all movement solutions that accomplish the task also load the tendon (e.g., calf raises or leg extensions). In addition, isolated exercises early in rehabilitation require less skill on the part of the individual and make it easier for the clinician to apply the correct intensity. More complex movement patterns can be incorporated into the program once there is a base tendon capacity established that is sufficient to afford movement solutions that direct sufficient load through that tendon.

### Time Under Tension and Relative Submaximal Lifting Capacity

The second key variable that HSR addresses is the rate of loading, which can be visualized as the slope of the force–time curve during the time interval from onset of movement to peak force. If loaded too rapidly, the tendon responds elastically with less strain and will adapt differently than when loaded at a slower rate [7]. In HSR a slow lifting tempo is used as a surrogate for controlling this rate of force development. Repetition speed has a significant impact on RSLC and is not controlled for in most of the available RSLC research. To control this parameter when examining the 1RM prediction tests, Reynolds et al. used a metronome with a 1-s eccentric and 1-s concentric tempo, i.e., 30 repetitions per minute (r/m), to pace their exercise [13]. This tempo is much quicker than most HSR studies, which take 6 s per repetition (10 r/m) or 8 s per repetition (7.5 r/m) [1–5].

The total time under tension (TUT) is defined as the time spent with the muscles under load and is determined by total repetitions and the speed of each repetition. However, TUT may be more of an artifact of the rate of movement used during a set than an important contributor to training effects. Instead, the duration of each repetition will affect the training intensity used if volume is held constant (i.e., lifting a maximum weight for the same number of repetitions but at different repetition durations). In addition, decreasing the speed of repetition (e.g., 4, 8, or 10 s) will significantly impact the number of repetitions that can be performed at the same load [14]. Wilk et al. found that exercise volume was lowest for sets using the slower repetition tempo, showing that even a small change in repetition tempo can significantly reduce the training volume. This was examined in the bench press at five different loads (40–80% 1RM) at tempos of approximately 2.8/1.4/1.0 s for the eccentric and concentric portion of the lift (5.6/2.8/1.9-s lifts) [15]. It was shown that slower repetition speeds drastically impact the RSLC with the weight lifted dropping below 70% RM at the sixth repetition for the slow group while the tempo was no longer maintained by the fourth repetition [15]. These data show that slower tempos at equivalent repetition zones reduce the intensity of the load that can be lifted. This reduction in RSLC appears to dip below the necessary 70% RM intensity threshold at around six repetitions when using a 6 s/repetition (s/rep) tempo. Since HSR protocols frequently use a 15/12/10/8 repetition approach at 6 s/rep, all of these repetition ranges exceed the probable maximum number that can be performed at the desired intensity. This strongly suggests that the intensity being used is not reaching the levels likely to elicit adaptations. Despite this, some good results have been seen with the HSR approach. This suggests that these outcomes are likely based on something besides adaptations to high load. It is interesting that Riel et al. were unable to replicate previous results for relief of plantar heel pain [5]. However, based on the very slow tempo (8 s/rep) used in their study, their discussion about under-dosage would make sense considering the impact tempo has on relative intensity at a set repetition range.

## Discussion and Conclusion

In conclusion, while slow tempo can be a practical cue to assist with coaching lower rates of lifting, these are not equivalent concepts and it may be important to balance slow lifting velocities with the impact they have on intensity. This is especially important for the clinician using a repetition-based goal of more than six repetitions while also using the commonly prescribed 6 s/rep tempo. Abandonment of these slower tempos is not the only way to maintain high intensity as this issue can be easily addressed in a few ways. If the slower repetition velocity is deemed important, it can be maintained while lowering the repetition goals and therefore increasing the intensity that can be utilized. There are established training approaches that balance these constraints, such as cluster training [16]. This approach keeps intensity high by programming in short intra-set rest periods, which allows incomplete recovery between small “micro sets” to allow more volume at a higher load. These “micro sets” are performed with a load of sufficient intensity and at volumes that allow their execution at the prescribed rates (e.g., 85% RM lifted for 3 × 3 reps with 15 s rest between each set of three) This allows for the accumulation of volume without sacrificing intensity by using strategic rest periods. This solution addresses the issues discussed in this paper without compromising any of the aspects currently deemed important for the exercise-based management of tendinopathy.

It is important to ensure that the goal of rehabilitation drives the process of rehabilitation. If the goal is adaptation due to heavier loads, then these considerations will be important for clinicians and researchers. While moving forward with the management of tendinopathy, it may be necessary to ensure that the “heavy” is put into the heavy slow resistance approaches.

## Declarations

### Availability of data and material

N/A.

### Code availability

N/A.
